# An alternative method to test scale invariance

**DOI:** 10.1016/j.mex.2020.100875

**Published:** 2020-03-20

**Authors:** Thitithep Sitthiyot, Pornanong Budsaratragoon, Kanyarat Holasut

**Affiliations:** a Department of Banking and Finance, Faculty of Commerce and Accountancy, Chulalongkorn University, Mahitaladhibesra Bld., 10th Fl., Phayathai Rd., Pathumwan, Bangkok 10330, Thailand; b Department of Chemical Engineering, Faculty of Engineering, Khon Kaen University, Mittapap Rd., Muang District, Khon Kaen 40002, Thailand

**Keywords:** Scale-free, Self-similarity, Rank-size distribution, Income and wealth, Lorenz curve, Kolmogorov–Smirnov test

## Abstract

We introduce an alternative method that is simple and can be used to test scale invariance or self-similarity in any types of data, irrespective of their distributions. Our method is based on estimating the Lorenz curve and the Kolmogorov–Smirnov test. This alternative method could be used as a preliminary screening before investigating further which types of distributions would fit the actual observations.

• We introduce a simple method to test scale invariance, regardless of data distributions.
• Our method is based on estimating the Lorenz curve and the Kolmogorov–Smirnov test.
• This alternative method could serve as an initial screening before investigating further which types of distributions would fit the actual data.

## Graphical abstract

**Figure:**
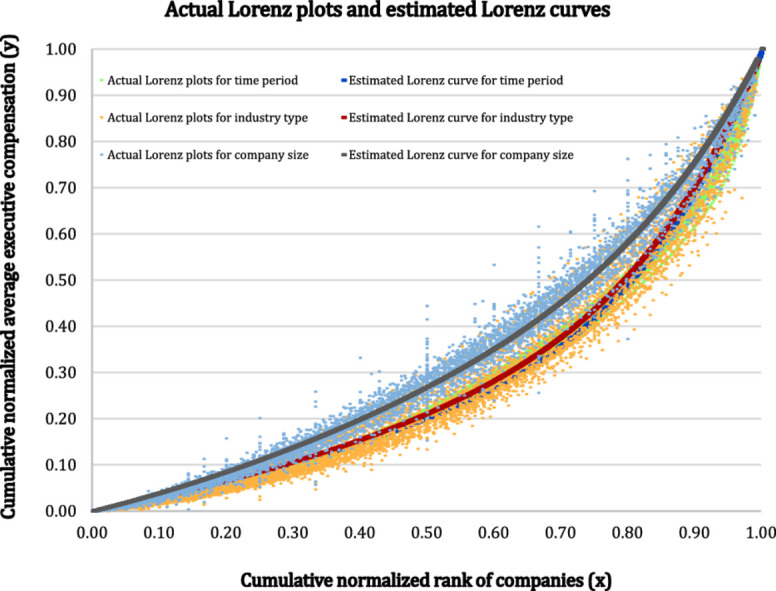

Specifications Table

**Table:** 

Subject Area:	Social Sciences
More specific subject area:	Rank-size distribution; Income and wealth distributions
Method name:	An alternative method to test scale invariance
Name and reference of original method:	Kolmogorov–Smirnov test. Retrieved from: http://www.physics.csbsju.edu/stats/KS-test.html. [Last accessed: 11 October 2019]. Lorenz, M. O. (1905). Methods of measuring the concentration of wealth. *Publ. Am. Stat. Assoc.*, 9 (70), pp. 209–219. Sitthiyot, T., Budsaratragoon, P., and Holasut, K. (2020). A scaling perspective on the distribution of executive compensation. *Physica A*., 543, 123556, https://doi.org/10.1016/j.physa.2019.123556.
Resource availability:	Sitthiyot, T. and Budsaratragoon, P. (2019). Data for: A scaling perspective on the distribution of executive compensation. *Mendeley Data*, v1, http://dx.doi.org/10.17632/4pxnf73xdt.1.

## Method details

### Method for testing scale invariance

Let z = quantity or size of thing of interest, x = cumulative normalized rank of z, and y = cumulative normalized value of z.

If the distribution of z is scale invariance, then it should look statistically self-similar when looked from different perspectives, dimensions, or different types of data grouping. The method for testing scale invariance or self-similarity in the distribution of z is based on estimating the Lorenz curve [1] and the Kolmogorov–Smirnov test [2]. It could be divided into four steps as follows:

*Step 1*. The method begins by sorting z into different groups. The sorted data for each group are then normalized and arranged in an ascending order. For each type of data groupings, the Cartesian coordinates are plotted where the abscissa is the cumulative normalized rank of z (x) and the ordinate is the cumulative normalized value of z (y). This would give us the actual Lorenz plots of normalized z for different types of data grouping.
*Step 2*. The next step is to fit a representative Lorenz curve to the actual Lorenz plots of normalized z for each type of data groupings. It should be noted that while there are many nonlinear functions that could be employed in order to fit the Lorenz curve, it should be aware that some nonlinear functions may result in the fitted Lorenz curve that is not realistic in a sense that not only does it not pass the coordinate (1, 1) but also gives us a negative value of cumulative normalized z (y) for a given value of cumulative normalized rank of z (x). To keep the method simple and yet realistic, the polynomial function is used to estimate the Lorenz curve on the conditions that the estimated Lorenz equation has to be an increasing function and must pass two coordinates which are (0, 0) and (1, 1). The curve fitting method is based on minimizing error sum of squares for each type of data groupings. This would give us equations for the estimated Lorenz curves, each of which represents the distribution of normalized z for different types of data grouping.
*Step 3*. By dividing the cumulative normalized value of z axis (the y-axis) into an equal number of bins and utilizing the coefficients obtained from the estimated equations for the Lorenz curves, for a given value of cumulative normalized z (y), we can calculate the value of cumulative normalized rank of z (x) and find out the proportion of population that falls in each of the corresponding bins on the cumulative normalized rank of z axis (the x-axis). Note that the number of equal bins could be varied according to the issues being investigated.
*Step 4*. The last step is to use the Kolmogorov–Smirnov test to compare whether or not the proportions of number of population that fall in the same bins on the cumulative normalized rank of z axis (the x-axis) calculated from different types of data grouping are statistically different from each other. It should be noted that the Kolmogorov–Smirnov test is non-parametric and commonly used to determine whether or not two datasets differ statistically. Its advantage is that it makes no assumption about the distribution of the data.

If the null hypothesis is not rejected, this would imply that the distribution of z, viewed from different dimensions, perspectives, or different types of data grouping, is statistically scale invariance or self-similar, and vice versa. This alternative method should serve as a preliminary screening before investigating further which distribution(s) would fit the actual observations.

### Method validation

To validate our method, we refer to our study [4]. In our study, we use annual data on average executive compensation of companies listed in the Stock Exchange of Thailand between 2002 and 2015 [3] to demonstrate our method. Average executive compensation is defined as the total executive compensation for each company divided by the number of executives in that company. Note that we use the average executive compensation since the Stock Exchange of Thailand does not collect the data on executive compensation for each individual executive. For this reason, the average executive compensation for a given company in any given year is used as a representative for individual executive compensation for that company in that year. We categorize the average executive compensation into three groups which are time period (T), industry type (I), and company size (C). Our study uses total assets as a proxy for company sizes. The data on the average executive compensation categorized by time period (T) and industry type (I) comprise 5922 observations while those grouped by size of company (C) contain 5089 observations. We would like to test whether or not the distributions of average executive compensation vary across three different types of data grouping.

In our study, scale invariance is defined as self-similarity of distributions of the average executive compensation across three types of data grouping which are time period (T), industry type (I), and company size (C). Let y(x)= the Lorenz function, where y= cumulative normalized average executive compensation and x= cumulative normalized rank of companies. If the distributions of the average executive compensation are scale invariance or y^T^(x) ≅ y^I^(x) ≅ y^C^(x), given 0  ≤  x  ≤  1, then the proportions of companies that fall in the same bin on the cumulative normalized rank of companies axis (the x-axis) should look statistically self-similar across time period (T), industry type (I), and company size (C). The following steps describe the method of testing scale invariance or self-similarity in the distribution of the average executive compensation.

**Fig. 1:**
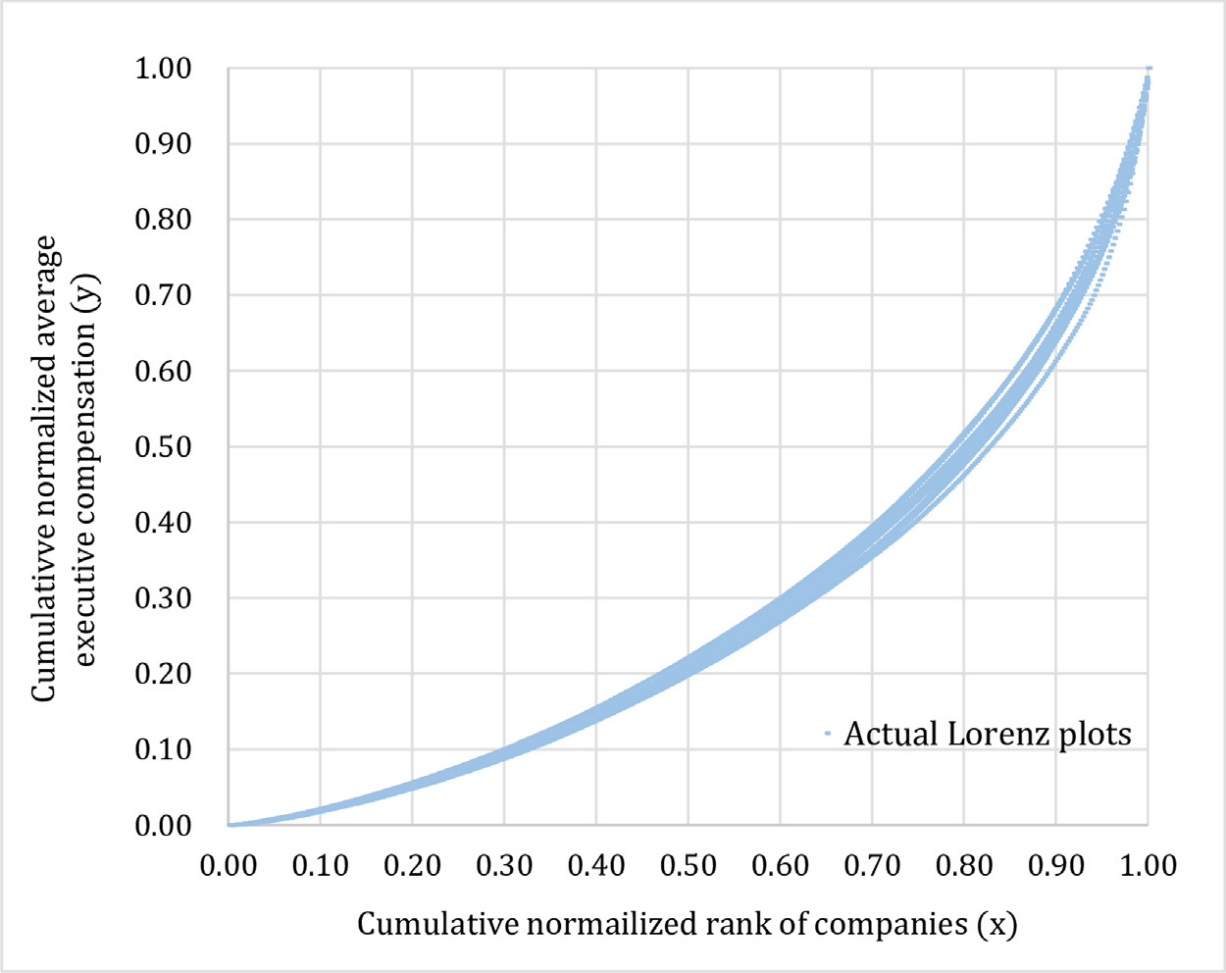
Actual Lorenz plots representing distributions of the average executive compensation classified by time period (2002–15).

**Fig. 2:**
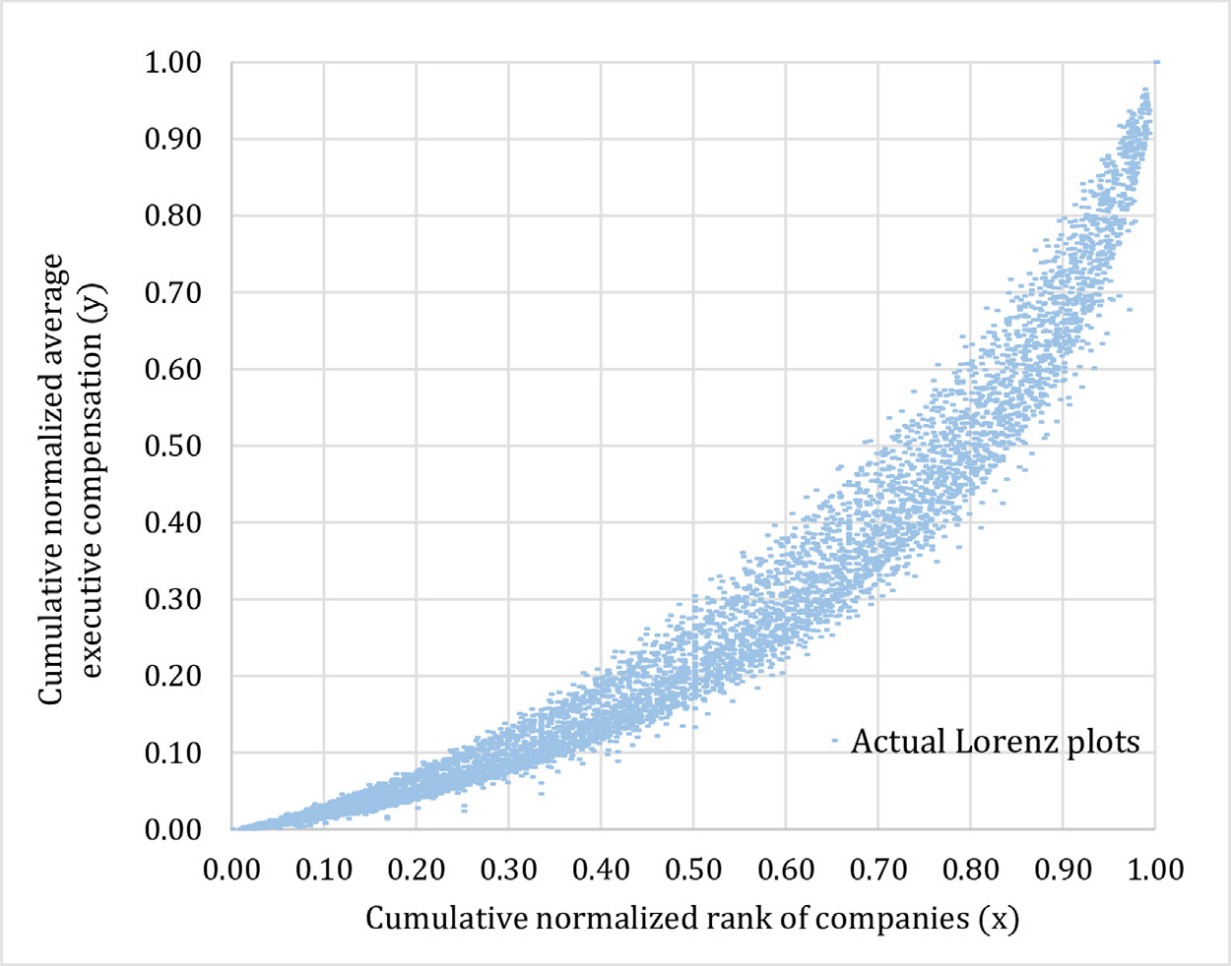
Actual Lorenz plots representing distributions of the average executive compensation classified by industry type (2002–15).

**Fig. 3:**
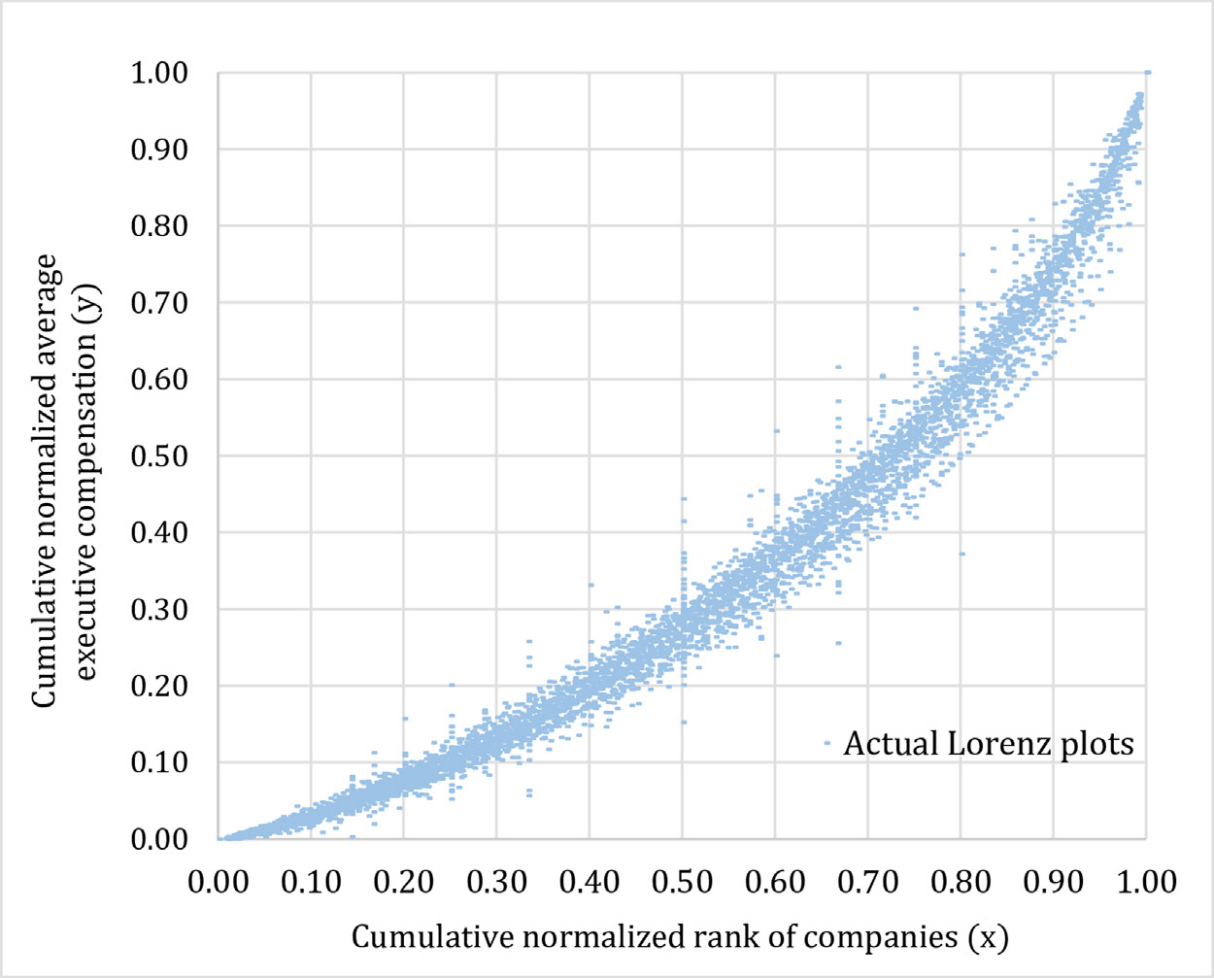
Actual Lorenz plots representing distributions of the average executive compensation classified by company size (2002–15).
*Step 2*. Fitting a representative Lorenz curve to the actual Lorenz plots for each type of data groupings. It should be noted that since our study pools data across years from 2002–15, before doing the curve fitting, it is necessary to test whether or not the distribution of the average executive compensation is relatively stable across time. This could be done by dividing the cumulative normalized average executive compensation axis (the y-axis) into twenty equal bins (0.00–0.05, 0.06–0.10, 0.11–0.15, …, 0.96–1.00) and counting the actual proportions of companies that fall in each corresponding bin (total of twenty bins) on the cumulative normalized rank of companies axis (the x-axis). As noted earlier, the number of equal bins can be adjusted accordingly depending upon the issues being investigated.

*Step 1*. Sorting the average executive compensation into three groups based on time period (T), industry type (I), and company size (C). The sorted data for each group are then normalized and arranged in an ascending order. For each type of data groupings, we plot the Cartesian coordinates where the abscissa is the cumulative normalized rank of companies (x) and the ordinate is the cumulative normalized average executive compensation (y). This would give us the actual Lorenz plots of normalized average executive compensation for three different types of data grouping according to time period (T), industry type (I), and company size (C) as shown in Figs. 1–3.

After obtaining the proportions of companies that fall in each corresponding bin (total of twenty bins) for all actual Lorenz plots, each of which representing each time period, we then conduct the pairwise Kolmogorov–Smirnov test to compare whether the proportions of companies that fall in the same bin (total of twenty bins) are statistically different from each other across time period or not. Due to the number of observations for time period (T) and industry type (I) is identical which is equal to 5922, just being sorted in different ways, we can simultaneously test whether or not the actual Lorenz plots that represent the distribution of the average executive compensation are statistically different across years for these two types of data categorization. For company size (C), we have to do a separate test since the number of observations is equal to 5089.

Tables 1 and 2 report the values of *D*-statistic from the pairwise Kolmogorov–Smirnov test which indicate that, for each type of data groupings, namely, time period (T), industry type (I), and company size (C), all actual Lorenz plots are not statistically different from each other across years with values of *D*-statistic between 0.00 and 0.20 and *p*-values, shown in parentheses, greater than 0.01 in all cases.

**Table 1:** Values of *D*-statistic from the pairwise Kolmogorov–Smirnov test show that, for time period (T) and industry type (I), there are no statistical differences in the distribution of average executive compensation across years with *p*-values, shown in parentheses, greater than 0.01 in all cases.

	2002	2003	2004	2005	2006	2007	2009	2010	2011	2012	2013	2014	2015
**2002**	0.00 (1.000)	0.10 (1.000)	0.15 (0.965)	0.10 (1.000)	0.05 (1.000)	0.05 (1.000)	0.10 (1.000)	0.10 (1.000)	0.15 (0.965)	0.20 (0.771)	0.10 (1.000)	0.10 (1.000)	0.10 (1.000)
**2003**		0.00 (1.000)	0.10 (1.000)	0.10 (1.000)	0.10 (1.000)	0.10 (1.000)	0.10 (1.000)	0.10 (1.000)	0.10 (1.000)	0.15 (0.965)	0.10 (1.000)	0.10 (1.000)	0.10 (1.000)
**2004**			0.00 (1.000)	0.10 (1.000)	0.10 (1.000)	0.10 (1.000)	0.10 (1.000)	0.10 (1.000)	0.10 (1.000)	0.15 (0.965)	0.10 (1.000)	0.10 (1.000)	0.10 (1.000)
**2005**				0.00 (1.000)	0.10 (1.000)	0.10 (1.000)	0.10 (1.000)	0.10 (1.000)	0.10 (1.000)	0.15 (0.965)	0.10 (1.000)	0.10 (1.000)	0.10 (1.000)
**2006**					0.00 (1.000)	0.05 (1.000)	0.15 (0.965)	0.10 (1.000)	0.15 (0.965)	0.20 (0.771)	0.10 (1.000)	0.15 (0.965)	0.15 (0.965)
**2007**						0.00 (1.000)	0.15 (0.965)	0.15 (0.965)	0.15 (0.965)	0.20 (0.771)	0.10 (1.000)	0.15 (0.965)	0.15 (0.965)
**2009**							0.00 (1.000)	0.05 (1.000)	0.10 (1.000)	0.10 (1.000)	0.10 (1.000)	0.10 (1.000)	0.10 (1.000)
**2010**								0.00 (1.000)	0.10 (1.000)	0.10 (1.000)	0.10 (1.000)	0.10 (1.000)	0.05 (1.000)
**2011**									0.00 (1.000)	0.10 (1.000)	0.10 (1.000)	0.05 (1.000)	0.05 (1.000)
**2012**										0.00 (1.000)	0.10 (1.000)	0.15 (0.965)	0.15 (0.965)
**2013**											0.00 (1.000)	0.10 (1.000)	0.05 (1.000)
**2014**												0.00 (1.000)	0.10 (1.000)
**2015**													0.00 (1.000)

**Table 2:** Values of *D*-statistic from the pairwise Kolmogorov–Smirnov test show that, for company size (C), there are no statistical differences in the distribution of average executive compensation across years with *p*-values, shown in parentheses, greater than 0.01 in all cases.

	2002	2003	2004	2005	2006	2007	2009	2010	2011	2012	2013	2014	2015
**2002**	0.00 (1.000)	0.15 (0.965)	0.15 (0.965)	0.15 (0.965)	0.10 (1.000)	0.10 (1.000)	0.15 (0.965)	0.15 (0.965)	0.15 (0.965)	0.20 (0.771)	0.15 (0.965)	0.20 (0.771)	0.20 (0.771)
**2003**		0.00 (1.000)	0.10 (1.000)	0.10 (1.000)	0.15 (0.965)	0.10 (1.000)	0.15 (0.965)	0.10 (1.000)	0.10 (1.000)	0.20 (0.771)	0.15 (0.965)	0.15 (0.965)	0.10 (1.000)
**2004**			0.00 (1.000)	0.10 (1.000)	0.15 (0.965)	0.15 (0.965)	0.10 (1.000)	0.10 (1.000)	0.10 (1.000)	0.15 (0.965)	0.10 (1.000)	0.10 (1.000)	0.15 (0.965)
**2005**				0.00 (1.000)	0.10 (1.000)	0.10 (1.000)	0.10 (1.000)	0.10 (1.000)	0.10 (1.000)	0.15 (0.965)	0.10 (1.000)	0.10 (1.000)	0.10 (1.000)
**2006**					0.00 (1.000)	0.10 (1.000)	0.15 (0.965)	0.15 (0.965)	0.10 (1.000)	0.20 (0.771)	0.15 (0.965)	0.15 (0.965)	0.15 (0.965)
**2007**						0.00 (1.000)	0.15 (0.965)	0.10 (1.000)	0.10 (1.000)	0.20 (0.771)	0.10 (1.000)	0.15 (0.965)	0.15 (0.965)
**2009**							0.00 (1.000)	0.05 (1.000)	0.10 (1.000)	0.15 (0.965)	0.10 (1.000)	0.10 (1.000)	0.05 (1.000)
**2010**								0.00 (1.000)	0.10 (1.000)	0.10 (1.000)	0.10 (1.000)	0.10 (1.000)	0.05 (1.000)
**2011**									0.00 (1.000)	0.10 (1.000)	0.10 (1.000)	0.10 (1.000)	0.10 (1.000)
**2012**										0.00 (1.000)	0.15 (0.965)	0.15 (0.965)	0.10 (1.000)
**2013**											0.00 (1.000)	0.05 (1.000)	0.05 (1.000)
**2014**												0.00 (1.000)	0.05 (1.000)
**2015**													0.00 (1.000)

**Fig. 4:**
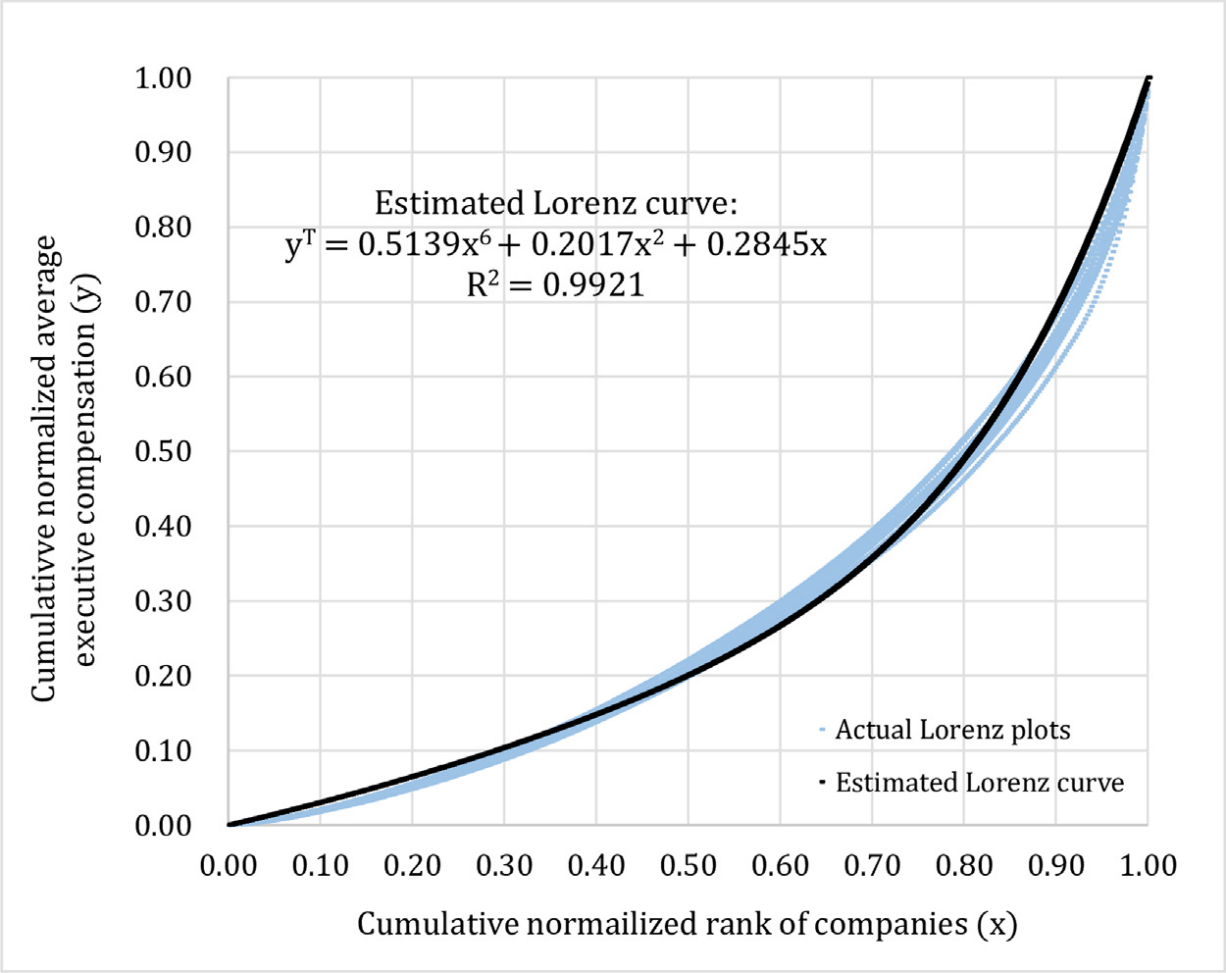
Estimated Lorenz curve for time period (2002–15).

**Fig. 5:**
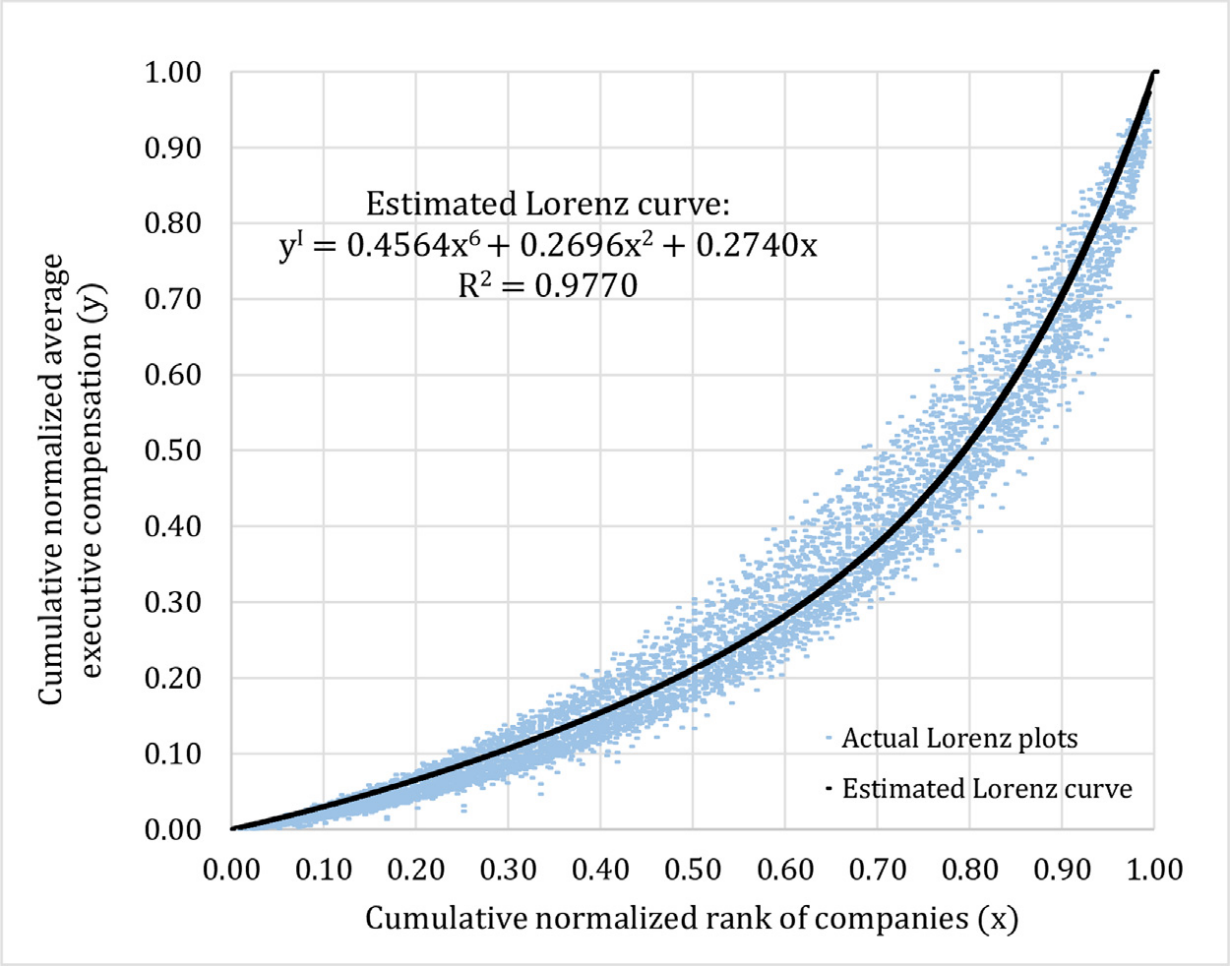
Estimated Lorenz curve for industry type (2002–15).

**Fig. 6:**
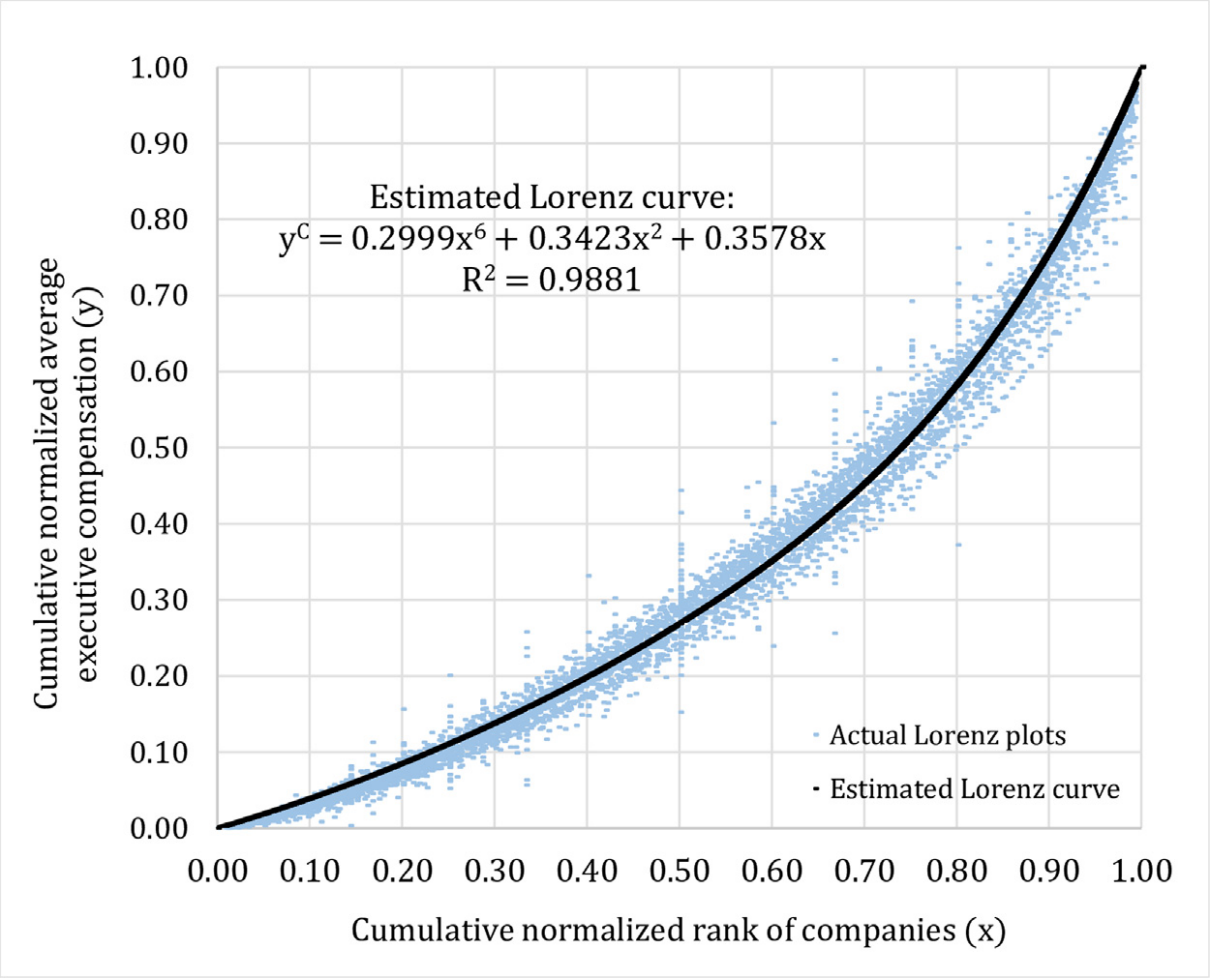
Estimated Lorenz curve for company size (2002–15).

Given these findings, we can proceed to pool the data across years and try to fit a representative Lorenz curve to the actual Lorenz plots for each type of data groupings. In our study, the polynomial function is used in order to estimate the Lorenz curve on the conditions that the estimated equation has to be an increasing function and must pass two coordinates which are (0, 0) and (1, 1). This curve fitting method based on minimizing error sum of squares is applied for each type of data groupings according to time period (T), industry type (I), and company size (C). The estimated equations for the Lorenz curve for three different types of data grouping could be described by the sixth degree polynomial functions as shown in Figs. 4–6.

*Step 3*. Dividing the cumulative normalized average executive compensation axis (the y-axis) into twenty equal bins (0.00–0.05, 0.06–0.10, 0.11–0.15, …, 0.96–1.00) and utilizing the coefficients obtained from the three estimated equations for the Lorenz curve as shown in Figs. 4–6 above, for a given value of cumulative normalized average executive compensation (y), we can work backward to calculate the value of cumulative normalized rank of companies (x) in order to find out the proportion of the listed companies that falls in each corresponding bin (total of twenty bins) on the cumulative normalized rank of companies axis (the x-axis) as shown in Table 3 below.

**Table 3:** Proportions of companies that are in the same bin (total of twenty bins) for three different types of data grouping and results from the Kolmogorov–Smirnov test.

Cumulative normalized average executive compensation (y)	Proportions of companies based on 3 different data groupings (x)			Kolmogorov–Smirnov test results (between 2 groups)		
	Time period	Industry type	Company size	Time period vs. industry type	Time period vs. company size	Industry type vs. company size
0.00–0.05	0.1580	0.1579	0.1248	*D*-statistic = 0.05 *p*-value = 1.000	*D*-statistic = 0.25 *p*-value = 0.497	*D*-statistic = 0.20 *p*-value = 0.771
0.06–0.10	0.1326	0.1265	0.1043			
0.11–0.15	0.1133	0.1066	0.0911			
0.16–0.20	0.0950	0.0903	0.0811			
0.21–0.25	0.0778	0.0759	0.0728			
0.26–0.30	0.0634	0.0635	0.0653			
0.31–0.35	0.0521	0.0534	0.0585			
0.36–0.40	0.0436	0.0454	0.0523			
0.41–0.45	0.0372	0.0391	0.0468			
0.46–0.50	0.0323	0.0342	0.0419			
0.51–0.55	0.0285	0.0302	0.0377			
0.56–0.60	0.0254	0.0271	0.0341			
0.61–0.65	0.0229	0.0244	0.0309			
0.66–0.70	0.0209	0.0223	0.0283			
0.71–0.75	0.0192	0.0205	0.0259			
0.76–0.80	0.0177	0.0189	0.0239			
0.81–0.85	0.0165	0.0176	0.0222			
0.86–0.90	0.0154	0.0164	0.0207			
0.91–0.95	0.0145	0.0154	0.0193			
0.96–1.00	0.0136	0.0145	0.0181
*Step 4*. Conducting the Kolmogorov–Smirnov test to compare whether or not the proportions of the listed companies that fall in the same bin (total of twenty bins) on the cumulative normalized rank of companies axis (the x-axis) calculated from three different types of data grouping are statistically different from each other. For example, as shown in Table 3, we would like to test whether the proportions of the listed companies that are in the first bin (0.00–0.05) for time period (0.1580), industry type (0.1579), and company size (0.1248) are statistically different from one another, and so on. Our results are reported in Table 3.

The Kolmogorov–Smirnov test results show that the difference between the distribution of proportions of companies categorized by time period vs. by industry type is statistically not significant with *p*-value = 1.000. The difference between the distribution of proportions of companies grouped by time period vs. by company size is also statistically not significant with *p*-value = 0.497. In addition, the difference between the distribution of proportions of companies sorted by industry type vs. by company size is statistically not significant with *p*-value = 0.771. The results from the Kolmogorov–Smirnov test indicate that there are no statistical differences among the distributions of proportions of companies according to three different types of data grouping since *p*-values are greater than 0.01 in all three cases. Our results indicate that the distributions of the average executive compensation, viewed from three different perspectives, are scale invariance or self-similar. In other words, time period, type of industry, and company size have no effects on the distributions of the average executive compensation.

### Author contributions

**Thitithep Sitthiyot:** Conceptualization, Methodology, Formal Analysis, Writing - Original Draft Preparation, Writing - Review and Editing. **Pornanong Budsaratragoon:** Investigation, Resource, Writing - Review and Editing. **Kanyarat Holasut:** Validation, Writing - Review and Editing.

## Declaration Competing of Interest

The authors declare no competing interests.
